# Combined effects of calcium‐alginate coating and *Artemisia fragrance* essential oil on chicken breast meat quality

**DOI:** 10.1002/fsn3.2856

**Published:** 2022-04-07

**Authors:** Kazem Alirezalu, Amir Hossein Moazami‐Goodarzi, Leila Roufegarinejad, Milad Yaghoubi, Jose M. Lorenzo

**Affiliations:** ^1^ Department of Food Science and Technology Ahar Faculty of Agriculture and Natural Resources University of Tabriz Tabriz Iran; ^2^ Department of Food Science and Technology Tabriz Branch Islamic Azad University Tabriz Iran; ^3^ 56947 Department of Food Science and Technology Faculty of Agriculture University of Tabriz Tabriz Iran; ^4^ Centro Tecnológico de la Carne de Galicia Parque Tecnológico de Galicia Ourense Spain; ^5^ Área de Tecnología de los Alimentos Facultad de Ciencias de Ourense Universidad de Vigo Ourense Spain

**Keywords:** aromatic plants, essential oils, meat preservation, microbial quality, natural compounds

## Abstract

The objective of the present study was to evaluate the effects of calcium‐alginate (CA) containing *Artemisia fragrance* essential oils (AFEOs) as a potential antioxidant and antimicrobial coating on quality attributes and shelf life of chicken meat throughout keeping period (4°C). Five treatments were produced as follows: T1 (distilled water as control), T2 (2% CA), T3 (2% CA +500 ppm AFEOs), T4 (2% CA +1000 ppm AFEOs), and T5 (2% CA +1500 ppm AFEOs). The chicken meats packaged in polyethylene bags at atmospheric condition and physicochemical, microbiological, and organoleptic properties were assessed at days 1, 4, 8, and 12. There was no remarkable difference in proximate composition (moisture, ash, protein, and fat) of meat samples by treating with CA or AFEOs. The results revealed that CA +AFEOs coating reduced significantly the pH, total volatile base nitrogen **(**TVB‐N), and thiobarbituric acid reactive substances (TBARS) values and also displayed higher contents of the total phenolic content (TPC) and redness value when compared with control. According to results, 2% CA +1500 ppm AFEOs reduced 58.3 (mg MDA (malondialdehyde)/kg) and 0.63 (mg/100 g) of TBARS and TVB‐N values when compared to control, respectively. The microbiological count showed that CA +AFEOs had a significantly higher inhibitory impact on the total viable count (TVC), coliforms, molds and yeasts. At day 12, 6.89 Log CFU (colony‐forming units)/g was recorded for TVC in 2% CA +1500 ppm AFEOs, which was the lowest overall. This treatment also displayed the reduction of 2.97 Log CFU/g in coliforms and 3.3 Log CFU/g in molds and yeasts in comparison with uncoated samples. The outcomes of pH, TBARS, TPC, color values, microbiological count, and organoleptic properties suggested 2% CA +1500 ppm AFEOs as an efficient coating for quality stability and improving the shelf life of chicken breast meat without negative impact on organoleptic properties.

## Graphical abstract

The objective of the present study was to evaluate the effects of calcium‐alginate (CA) containing *Artemisia fragrance* essential oils (AFEOs) as a potential antioxidant and antimicrobial coating on quality attributes and shelf life of chicken meat throughout keeping period (4°C). The outcomes of pH, thiobarbituric acid reactive substances (TBARS), total phenolic content (TPC), color values, microbiological count, and organoleptic properties suggested 2% CA +1500 ppm AFEOs as an efficient coating for quality stability and improving the shelf life of chicken breast meat without negative impact on organoleptic properties.

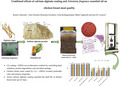

## 1 INTRODUCTION

Chicken meat due to a high amount of protein, moisture, and pH values is a susceptible place for oxidation reactions and microbiological contamination by pathogenic bacteria such as *Campylobacter jejuni*, *Listeria monocytogenes*, *Salmonella* spp., and *Escherichia coli* which lead to low shelf life (Wickramasinghe et al., 2019; Yaghoubi et al., 2021). However, the main challenge of researchers and also food industries is in improving the quality stability of fresh chicken meat. For extending the shelf life and quality stability of food products, particularly of meat and meat products, edible coatings and films containing plant extracts and essential oils are known as a novel and potential approach (Gutirerrez‐García et al., 2021; Javaherzadeh et al., 2020; Karimian et al., 2019; Shahhoseini et al., 2019; Shahosseini et al., 2021). Compared with conventional packaging like modified atmosphere packaging (MAP), vacuum packaging, the edible coatings due to their ability to block carbon dioxide, oxygen, and water vapor from outside and impede the moisture loss, could directly contact the meat to maintain the quality and prolong the shelf life (Xiong et al., 2020).

Polysaccharides like alginate due to particular properties, such as good film‐forming, thickening, and gel‐producing capability, are utilized widely as biopolymer films or coating components (Draget et al., 2005). Calcium‐alginate because of its high biodegradability, availability, and low cost is an efficient alternative for other natural coatings in the meat industry (Hassan et al., 2018). Surendhiran et al. (2019) reported a significant increase in chicken meat shelf life by using alginate/polyethylene oxide (PEO) containing phlorotannin.

Natural plant extracts and essential oils (EOs) have been widely utilized to improve the shelf life and quality stability of meat and meat products (Bagheri et al., 2016; Javadian et al., 2017; Safari et al., 2018). The genus *Artemisia* as a perennial plant that belongs to the Asteraceae family (with more than 500 species) and is widely found in Russia, Iran, and their neighboring regions (Bora & Sharma, 2011). *Artemisia fragrance* with high antibacterial and antioxidant activities (Jaradat et al., 2022; Yaghoubi et al., 2021) is a rich source of 1,8‐cineole, α‐thujone, β‐thujone, and camphor (Jaradat et al., 2022). There are many researches which have reported the potential antioxidant and antimicrobial properties of *Artemisia* in meat and meat products such as thigh and breast muscles in broilers (Wan et al., 2016), *Scomberoides commersonnianus* fillets (Farsanipour et al., 2020), and chicken meat (Yaghoubi et al., 2021). However, the synergistic impacts of calcium‐alginate (CA) with *Artemisia fragrance* essential oils (AFEOs) were not reported. The objective of the present work was to evaluate the preservative impacts of CA coating incorporated with *Artemisia fragrance* in chicken breast meat by evaluating the microbiological count and quality stability during storage period (4°C).

## 2 MATERIALS AND METHODS

### 2.1 Preparation of *Artemisia fragrance* essential oil (AFEOs)

The AFEOs were prepared by using the Clevenger‐type apparatus, 1 L water +400 g dry material of plant subjected to hydro‐distillation. The essential oils (EOs) were protected in dark glass bottles, wrapped with aluminum foil around it, and kept in a refrigerator (4°C) until used in chicken samples (1 day).

### 2.2 AFEOs compounds’ isolation

The AFEOs composition was analyzed by using gas chromatographic–mass spectrometric (GC–MS) apparatus (Varian, mod. Saturn 2100T, San Fernando, CA, USA). Helium (as the carrier gas; 1 cm^3^/min) and fused‐silica capillary column (50 m × 0.22 mm, 0.25‐μm film thickness) were utilized for the separation of EO components. The temperatures of detector and injector (splitless 20 cm^3^/min) were set at 260 and 280°C, respectively. From 50°C, the oven condition was increased (at the rate of 2°C/min) to 250°C and kept for 1 h. Compared to peak retention time of standard fatty acid methyl ester (FAMEs), the FAMEs of samples were analyzed and the peak area expressed as the compound percentage (Baldino et al., 2017).

### 2.3 Preparation of chicken meats

Three separate chicken breast meat (skinless, boneless, and weighed between 2 and 2.5 kg) were purchased from a local slaughterhouse (Azar morgh, EA, Iran) during three successive days (with three different preparations) and transferred to laboratory in ice boxes (five treatments ×three times of sampling with three sampling points for each sample). The chicken meat samples were sized 3 × 3 × 3 cm by a sterile knife. Twenty grams of sodium alginate (Keltone LV, ISP, San Diego, CA, USA) and calcium chloride solution (20 µg/ml) were added to an Erlenmeyer flask and reached 1000 ml by using distilled water (60°C), stirred on a heater (60°C) to homogenize the suspension using a magnetic stirrer, and cooled to room temperature before using the meat samples. The meat samples were treated according to: distilled water as control (T1), 2% CA (T2), 2% CA +500 ppm AFEOs (T3), 2% CA +1000 ppm AFEOs (T4), and 2% CA +1500 ppm AFEOs (T5). All meat samples were immersed in produced solutions for 1 h (at 4°C) and finally the samples were drained, packaged in polyethylene bags, and kept at 4°C for the evaluation of proximate composition, pH, color indices, TBARS, TVB‐N, total phenolic content (TPC), organoleptic properties, and microbiological count during days 1, 4, 8, and 12 of keeping time.

### 2.4 Proximate composition and pH value

The ash and moisture content of chicken samples was measured according to AOAC (Association of Official Analytical Chemists) (2006). The Soxhlet extraction technique was utilized for evaluation of the samples’ fat content (AOAC, 2006). The protein content of samples was measured by using the Kjeldahl method (AOAC, 2006). The meat samples’ pH was measured by using the Bozkurt and Erkmen (2007) method as follows: the meat samples (10 g) were homogenized in distilled water (100 ml) and assessed with calibrated pH meter (Hanna, Metrohm, Switzerland).

### 2.5 Measurement of thiobarbituricacid reactive substances (TBARS)

According to Eymard et al. (2005), the TBARS values of meat samples were measured by a spectrophotometer (Hitachi, Ltd., Tokyo, Japan) in triplicate at 532 nm. The compound 1,1,3,3 tetraethoxypropane (molecular weight (MW): 220.31 g/mol, Sigma‐Aldrich, Darmstadt, Germany) at different concentrations (0, 2, 4, 6, 8, and 10 ppm) was utilized for attaining the standard curve and the results were expressed as mg malondialdehyde (MDA)/kg of samples.

### 2.6 Determination of the total volatile base nitrogen (TVB‐N)

According to Goulas and Kontominas (2005), the meat samples’ TVB‐N values were analyzed by the Kjeldahl apparatus with a vapor distillation. The results were reported as mg nitrogen/100 g meat samples.

### 2.7 Total phenolic content (TPC)

The TPC of chicken meat samples was measured according to Liu et al. (2009) by the Folin–Ciocalteu reagent (Merck, Darmstadt, Germany) assay as follows: 100 ml of boiled distilled water and 50 g of meat were mixed together and kept for 20 min at room temperature. The solution was cooled, filtered, and mixed with saturated sodium carbonate solution (5 ml) and Folin–Ciocalteau reagent (2.5 ml). The solution was vortexed and kept (for 1 h) at a dark place. The TPC of samples was measured by ultraviolet–visible (UV–vis) spectrophotometer at 700 nm (Hitachi U‐3210; Hitachi, Ltd.). The standard curve was prepared by gallic acid (GA) (Merck) and the data were expressed as mg/100 g of gallic acid equivalent (GAE).

### 2.8 Determination of color values

The lightness (L^*^), redness (a^*^), and yellowness (b*) of chicken meat surfaces (both internal and external surfaces) were analyzed by a simple digital imaging system in triplicate (Leon et al., 2006). The meats were cut into 3 × 3 × 1 cm thickness for color assessment. For capturing images, digital camera (16 megapixels) was utilized under suitable light at room temperature (25°C) before instrument calibration with standard plates. Finally, the images were assessed by MATLAB software (The MathWorks, Inc., Natick, MA, USA) for the evaluation of L*, a*, and b* values of meat samples.

### 2.9 Microbiological analysis

Twenty‐five grams of chicken meat and 225 ml of 0.1% (w/v) peptone water (Difco, Becton Dickinson, Bergen County, New Jersey, USA) were mixed together by a sterile lab‐blender for 3 min (Neutec; Paddle Lab Blender, Albuquerque, New Mexico, USA). Peptone (0.1%) water was utilized for serial dilution preparation. Total viable count (TVC), coliforms, yeasts and molds were determined by PCA (plate count agar; Merck), VRB (violet red bile agar; Merck), and DRBC (dichloran rose‐bengal chloramphenicol agar; Merck), respectively, by the pour‐plate technique. The incubation time for TVC, coliforms, yeasts and molds was 30°C for 48–72 h, 37°C for 24 h, and 25°C for 5 days, respectively. The results were expressed as Log CFU/g of meat (FDA, 2013).

### 2.10 Sensory properties

A total of 72 persons (48 females and 24 males) aged 20–35 years were selected as panelists who had prior experience about chicken breast meat for evaluation of sensory attributes in six booths with 12 panelists for each booth. A randomized (complete) block design was used for estimating the odor, color, freshness, texture, and overall acceptability of meats. The chicken meat samples were individually labeled with stochastic numbers and randomly tested under controlled light and temperature. The odor, color, freshness, and texture were scored according to the hedonic scale (1: *really dislike*, 5: *really like*) and the averages of scores reported as the overall acceptability score for each group (Economou et al., 2009; Stone & Sidel, 2004).

### 2.11 Statistical analysis

The experimental results with five treatments ×four time periods ×three repetition ×three runs were analyzed according to statistical software SAS (v.9; SAS Institute Inc., Cary, NC, USA). Variance homogeneity and normal distribution had been determined previously (Shapiro–Wilk). The pH, TPC, color indices, TBARS values, TVB‐N, organoleptic attributes, and microbiological counts were evaluated through random block design (considering a mixed linear model), including treatments and refrigerated period as fixed effects, and replicate as a random effect. Proximate composition of meat samples including protein, ash, fat, and moisture was also assessed through analysis of variance (ANOVA) (*p *< .05), followed by Tukey's test. All data in figures and tables were reported as mean values ±*SE*.

## 3 RESULT AND DISCUSSION

### 3.1 Gas chromatography–mass spectrometry analysis

The AFEOs volatile chemical compounds are presented in Table 1. The data indicated that with 40.23%, thujone displayed the highest amount followed by 1,8‐cineole, L‐camphor, and isobornyl alcohol with 21.06%, 11.88%, and 3.47%, respectively. According to obtained results, 99.39% of total AFEOs volatile component were identified. The results of the present work are in agreement with those reported by Yaghoubi et al. (2021). The authors evaluated the chemical compounds of AFEOs and reported similar results. In stark contrast, Baldino et al. (2017) showed some different contents for AFEOs volatile component. Variations in cultivation, extraction conditions, plant organs, stage of maturity, cultivars, soil composition, genetic as well as climate conditions could be the main reasons for these disagreements (Baldino et al., 2017).

**TABLE 1:** *Artemisia fragrance* essential oils (AFEOs) composition used for samples treatment

Name	Essential oil components area (%)
4‐Carene	0.44
Methyl cinnamate	0.23
3‐Carene	0.21
β‐Cymene	1.35
p‐Cymene	0.46
Camphene	0.9
cis‐Salvene	0.21
l‐Phellandrene	0.46
Sabinene	0.44
α‐Terpinolene	0.74
α‐Pinene	0.2
β‐Phellandrene	0.50
β‐Pinene	0.21
γ‐Terpinene	0.7
Verbenene	0.16
1,8‐Cineole	21.06
4‐Terpineol	2.67
L‐Camphor	11.88
cis‐Jasmone	0.52
Isobornyl alcohol	3.47
L‐Carvone	1.14
Myrtenal	0.15
Myrtenol	2.15
Pinocarvone	0.24
Piperitone	0.99
Sabinyl acetate	1.65
Thujone	40.23
Sesquiterpenes (STs)	0.38
Germacrene‐D	0.38
Copaene	0.37
Oxygenated sesquiterpenes (OSTs)	2.05
Carvacrol	1.11
cis‐Davanone	0.95
Others (OTH)	0.40
1‐Octen−3‐ol	0.44

### 3.2 Proximate composition and pH values

Physicochemical attributes of coated chicken meats with CA containing AFEOs are presented in Table 2. The content of moisture, fat, ash, and protein ranged between 75.69%–75.99%, 1.38%–1.44%, 1.13%–1.19%, and 21.21%–21.51%, respectively. The chemical composition of samples was not affected by CA +AFEOs coatings. The results of the present work are in agreement with those of Agregan et al. (2019), Alirezalu, Pirouzi, et al. (2021), and De Carvalho et al. (2019) on pork patties, beef fillet, and lamb patties, respectively. The authors indicated that proximate composition was not affected by added natural antioxidant.

**TABLE 2:** Proximate composition of meat samples treated with calcium‐alginate +Artemisia *fragrance* essential oils (AFEOs) throughout keeping time at 4°C

Chicken samples	Properties^a^ (%)			
	Moisture	Fat	Ash	Protein
T1	75.84	1.42	1.19	21.37
T2	75.72	1.39	1.17	21.51
T3	75.91	1.40	1.13	21.21
T4	75.78	1.44	1.15	21.34
T5	75.69	1.38	1.14	21.41
*SEM*	0.867	0.085	0.019	0.237

Note

T1: Control, T2: 2% CA, T3: 2% CA +500 ppm AFEOs, T4: 2% CA +1000 ppm AFEOs, and T5: 2% CA +1500 ppm AFEOs.

^a^ There was no significant difference of chemical properties among treatment groups and the control.

**FIGURE 1:**
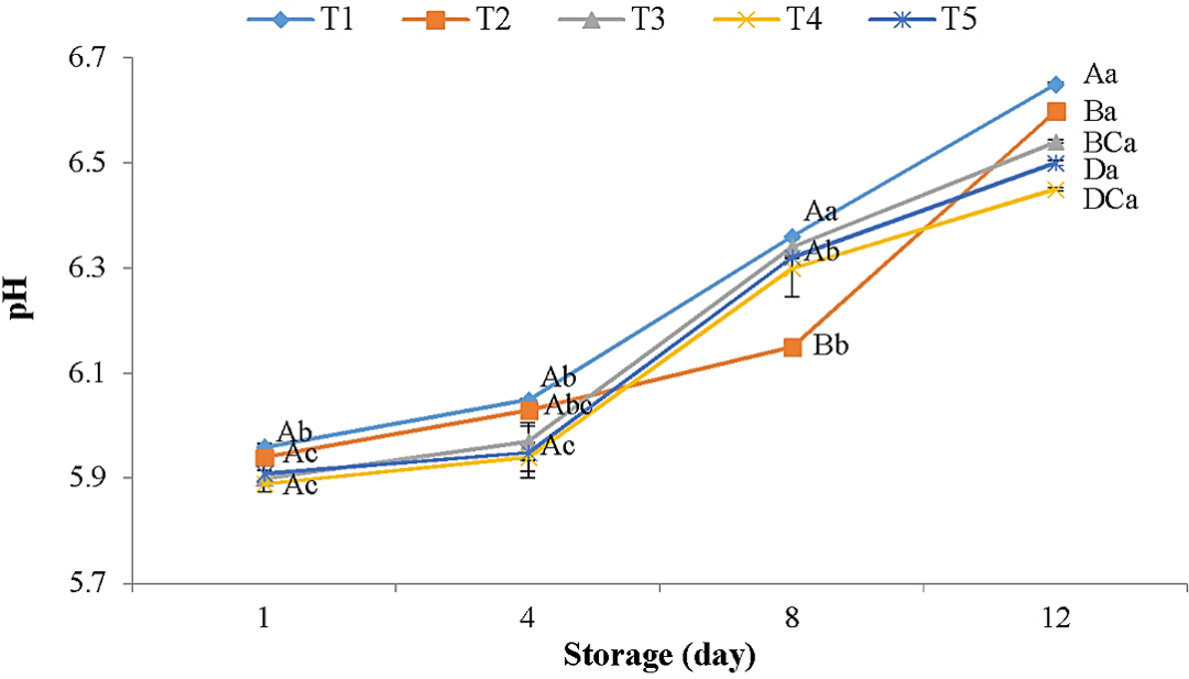
pH changes in chicken meat samples treated with calcium‐alginate (CA) combined with *Artemisia fragrance* essential oils (AFEOs) throughout keeping time at 4°C. T1: Control, T2: 2% CA, T3: 2% CA +500 ppm AFEOs, T4: 2% CA +1000 ppm AFEOs, and T5: 2% CA +1500 ppm AFEOs. ^a–c^ Different lowercase letters throughout storage indicate significant (*p* < .05) differences. ^A–D^ Different capital letters between meat samples indicate significant (*p* < .05) differences

The pH value, which is usually below 6 in fresh meat, can potentially affect the shelf life of meat by changing microbial levels and bacteriostatic function (Alirezalu et al., 2021; Cullere et al., 2018). The pH values of samples increased throughout refrigerated time and the rate of this increase was remarkably higher in control (Figure 1). Accumulation of alkaline components and production of basic nitrogenous components by the psychrotrophic bacteria proteolytic activity and autochthonous enzymes’ autolytic activity throughout keeping time can lead to increase in pH values (Radha krishnan et al., 2014; Wang et al., 2017). The antimicrobial activities of AFEOs lead to suppression of the increase in pH values in coated samples when compared to control. The TVB‐N results of the present work also paralleled pH changes which showed that AFEOs have potential antimicrobial activity against psychrotrophic bacteria, especially *Pseudomonas* spp., and lead to safety and high shelf life of meat samples.

The application of CA +AFEOs coatings resulted in lower pH values than reduction of microorganisms’ growth by new coatings on the meat surface. This phenomenon could be attributed to the presence of AFEOs in CH coating that leads to a reduction in the permeability of carbon dioxide produced by microbial activity, as a result of carbon dioxide accumulation in meat and pH decrement which can in turn be effective on meat microbial count reduction. The results of microbial growth in Table 5 are also in agreement with pH results. The Liu et al. (2019), Yaghoubi et al. (2021), and Zhang et al. (2021) reported a similar trend for pH values in chilled meat, chicken meat, and beef by treating natural preservatives.

### 3.3 Determination of thiobarbituricacid reactive substances (TBARS) and total volatile base nitrogen (TVB‐N)

One of the most vital indicators for the measurement of lipid oxidation is the TBARS values, which revealed the secondary products (particularly aldehydes) as a result of lipid oxidation (Cai et al., 2014; Sun et al., 2019). The impacts of CA coating in combination with AFEOs are presented in Table 3. The TBARS values of all meat samples were remarkably increased throughout keeping time (particularly in control). Liu et al. (2019), Jonaidi Jafari et al. (2018), Pabast et al. (2018), and Fang et al. (2018) used natural coatings +natural plant extract in TBARS values of chilled meat, lamb meat, fresh pork, and chicken fillets, respectively, and reported the similar results with the present work. Coating meat samples leads to a low oxygen availableness of meat surfaces which may be the main reason for the low TBARS values in coated samples (Sogut & Seydim, 2019).

**TABLE 3:** The thiobarbituricacid reactive substances (TBARS) and total volatile base nitrogen (TVB‐N) values of meat samples treated with calcium‐alginate +AFEOs (*Artemisia fragrance* essential oils) throughout keeping time at 4°C

Parameters	Treatments	Storage (day)			
		1	4	8	12
TBARS (mg MDA/kg)	T1	0.32 ± 0.005^Ad^	0.51 ± 0.009^Ac^	0.95 ± 0.067^Ab^	1.37 ± 0.005^Aa^
	T2	0.23 ± 0.007^Ad^	0.47 ± 0.003^Ac^	0.86 ± 0.009^ABb^	1.29 ± 0.005^Aa^
	T3	0.20 ± 0.019^Ad^	0.44 ± 0.075^ABc^	0.83 ± 0.009^ABb^	0.99 ± 0.066^Ba^
	T4	0.20 ± 0.007^Ad^	0.42 ± 0.011^ABc^	0.67 ± 0.023^Bb^	0.95 ± 0.036^Ba^
	T5	0.17 ± 0.003^Ad^	0.26 ± 0.003^Bc^	0.54 ± 0.019^Cb^	0.74 ± 0.046^Ca^
TVB‐N (mg/100 g)	T1	6.00 ± 0.80^Ad^	10.20 ± 0.80^Ac^	48.00 ± 0.01^Bc^	90.70 ± 0.40^Aa^
	T2	5.53 ± 0.46^Ad^	10.20 ± 0.80^Ac^	45.90 ± 0.21^Ab^	58.43 ± 0.80^Ba^
	T3	4.60 ± 0.01^ABd^	8.80 ± 0.01^ABc^	45.20 ± 0.61^Ab^	55.70 ± 0.40^Ba^
	T4	3.90 ± 0.40^BCc^	7.40 ± 0.80^BCb^	39.60 ± 0.80^Ba^	40.36 ± 0.34^Ca^
	T5	2.83 ± 0.23^Cd^	6.80 ± 0.80^Cc^	25.20 ± 1.61^Cb^	32.40 ± 0.01 Da

Note

T1: Control, T2: 2% CA, T3: 2% CA +500 ppm AFEOs, T4: 2% CA +1000 ppm AFEOs, and T5: 2% CA +1500 ppm AFEOs. ^a–d^ Different lowercase letters throughout storage indicate significant (*p* < .05) differences ^A–D^ Different capital letters between meat samples indicate significant (*p* < .05) differences.

Similarly, Alizadeh Behbahani et al. (2017) and Pabast et al. (2018) reported that coating when applied directly to the meat surface may serve as a barrier. Consequently, this reduces the diffusion of oxygen into the meat surface and retards lipid oxidation.

Moreover, high antioxidant properties of AFEOs can also lead to low oxidation throughout keeping period (Yaghoubi et al., 2021). Based on TBARS values, the CA coating in combination with AFEOs can extend the chicken meat's shelf life due to its good antioxidant properties. The results of TBARS values revealed that the presence of AFEOs in the coating could inhibit lipid oxidation. The antioxidant properties of EOs in edible coatings on meat stability have been well documented.

TVB‐N values of samples, which are one of the important indicators in meat and meat products’ shelf life (Ala & Shahbazi, 2019), are presented in Table 3. At day 1, the TVB‐N values of samples ranged from 2.83 to 6 mg N/100 g which was the allowable situation for chicken meat. The TVB‐N values were significantly (*p *< .05) increased throughout storage in all samples, particularly in control. The TVB‐N values of 58.43, 55.70, 40.36, and 32.40 mg/100g were achieved in treated samples with 2% CA (T2), 2% CA +500 ppm AFEOs (T3), 2% CA +1000 ppm AFEOs (T4), and 2% CA +1500 ppm AFEOs (T5), respectively, by contrast 90.70 mg/100 g (T1) for control samples at day 12. The results also indicated that the TVB‐N values increase more slowly by increasing the concentration of AFEOs. The microbiological and TVB‐N values of meat samples are paralleled. Abdou et al. (2018) and MojaddarLangroodi et al. (2018) reported a similar trend in TVB‐N reduction at chicken fillets and meat coated with natural coating combined with natural extracts and essential oils. Lower microbial growth and oxidation reactions in treated samples might be the principal reasons for low TVB‐N values. There was a high correlation with pH and TVB‐N in all meat samples during storage which showed an increase in proteolytic bacteria count and their enzymes’ autolytic activity throughout refrigerated storage can lead to increase in free amine compounds, volatile nitrogenous compounds, and subsequently in pH and TVB‐N values (Alirezalu et al., 2021).

### 3.4 Total phenolic content (TPC)

**FIGURE 2:**
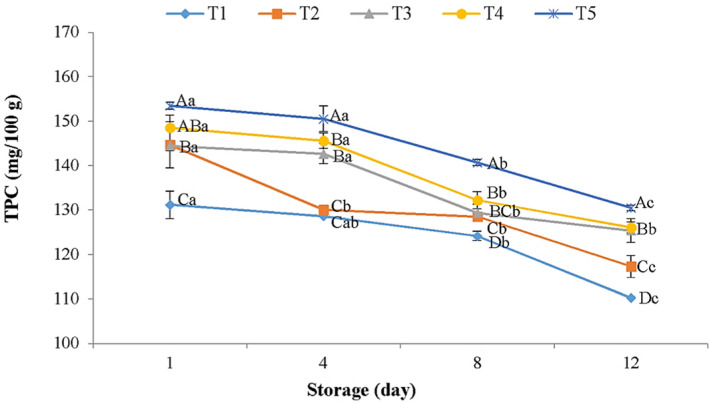
Total phenolic content (TPC) of chicken meat samples treated with calcium‐alginate (CA) combined with *Artemisia fragrance* essential oils (AFEOs) throughout keeping time at 4°C. T1: Control, T2: 2% CA, T3: 2% CA +500 ppm AFEOs, T4: 2% CA +1000 ppm AFEOs, and T5: 2% CA +1500 ppm AFEOs. ^a–c^ Different lowercase letters throughout storage indicate significant (*p* < .05) differences. ^A–D^ Different capital letters between meat samples indicate significant (*p* < .05) differences

The natural compounds such as plant extracts and EOs are rich source of phenolic components renowned for their antioxidant, antimicrobial, and functional attributes (Alirezalu, Hesari, et al., 2021; Alirezalu et al., 2020; Zhang et al., 2021). The results illustrated in Figure 2 show the impacts of CA in combination with AFEOs on TPC during keeping time intervals. The TPC decreased during the storage period and the rate of this decrease was remarkably higher in control samples. Oxidation reactions throughout keeping time may be the main reason for the decreasing phenolic content among meat samples (Daskalaki et al., 2009; Nemati et al., 2020). The TPC of chicken meats coated with CA +AFEOs was significantly (*p *< .05) (117.32–130.47 mg GA (gallic acid equivalent)/100 g DM (dry matter)) higher than that of control samples (110.28 mg GA/100 g DM). The presence of high phenolic components in plant EOs (Nemati et al., 2021; Shirzad et al., 2021) might be the principal reason for the high TPC in coated meat samples. Alirezalu, Hesari, et al. (2021) and Liu et al. (2009) also reported similar results on treated frankfurter‐type sausages and fresh chicken sausages by natural plant extracts, respectively.

### 3.5 Determination of color values

The lightness (L*), redness (a*), and yellowness (b*) of chicken meat samples were remarkably (*p *< .05) affected by both coating materials and keeping time (Table 4). The lightness in all meat samples was decreased throughout storage time and at the end of storage time the control samples displayed significantly (*p *< .05) the lowest L^*^ values (50.05) when compared to others. The coated samples with 2% CA +1500 ppm AFEOs displayed significantly the highest (60.50) amount of lightness at day 12. Higher L* values in coated samples in compared to control might be caused by barrier and antioxidant properties of CA and AFEOs. A similar trend for lightness was also reported by Alirezalu et al. (2019) on sausages treated by chitosan +natural plant extracts. The reduction in a* values throughout keeping period may be attributed to production of met‐myoglobin by oxidation reactions (Lorenzo et al., 2014; Zhang et al., 2021). Treated samples with CA +AFEOs displayed higher a* in comparison with control which could be attributed to antioxidant properties of coating. Direct exposure of meat to oxygen potentially leads to oxidation of myoglobin to met‐myoglobin which might be the main reason for the darker color change of uncoated samples during refrigeration compared with coated samples. Hence, coating acted as a barrier for the oxygen in environment and the meat samples, effectively gave rise to low myoglobin oxidation, particularly in coatings with a higher amount of EOs, in which *Artemisia* EOs could delay by partially depleting oxygen during the occurrence of oxidation in myoglobin. De Carvalho et al. (2019) evaluated color stability and reported similar trends for a* in lamb burgers treated with natural plant extracts. Enzymatic browning reaction of phenolic compounds potentially influenced the b* values. However, treated samples with CA +AFEOs and control displayed the lowest and highest amounts of the browning reaction, respectively.

**TABLE 4:** Evaluation of color values of meat samples treated with calcium‐alginate (CA) + *Artemisia fragrance* essential oils (AFEOs) throughout keeping time at 4°C

Parameters	Treatments	Storage (day)			
		1	4	8	12
	T1	57.43 ± 0.37^Ca^	56.01 ± 0.75^Ca^	55.28 ± 0.37^Ca^	50.05 ± 1.13^Cb^
L^*^	T2	56.77 ± 1.96^Ca^	56.28 ± 0.37^Ca^	56.77 ± 0.3^Ca^	50.74 ± 0.37^Cb^
	T3	60.05 ± 1.13^Ba^	60.05 ± 1.13^Ba^	57.42 ± 1.12^Cb^	56.16 ± 0.75^Bb^
	T4	65.28 ± 0.37^Aa^	64.63 ± 0.75^Aa^	60.88 ± 0.37^Bb^	55.88 ± 0.37^Bc^
	T5	67.43 ± 0.37^Aa^	65.76 ± 1.1^Aa^	63.32 ± 0.75^Aab^	60.50 ± 0.37^Ab^
	T1	11.13 ± 0.82^Ba^	9.70 ± 1.09^Cab^	8.75 ± 0.36^BCbc^	7.81 ± 0.11^CDc^
a^*^	T2	13.98 ± 0.27^ABa^	11.60 ± 0.01^BCb^	7.80 ± 0.3^Cc^	6.38 ± 0.27^Dc^
	T3	14.03 ± 0.27^Aa^	12.55 ± 0.01^ABb^	7.33 ± 0.27^Cc^	7.80 ± 0.54^CDc^
	T4	14.93 ± 0.82^Aa^	13.03 ± 0.27^Ab^	9.23 ± 0.82^Bc^	8.75 ± 1.09^BCc^
	T5	15.40 ± 0.36^Aa^	14.65 ± 0.54^Aa^	13.23 ± 0.27^Ab^	12.01 ± 0.54^Ab^
	T1	18.52 ± 0.01^Aab^	17.55 ± 0.27^ABa^	17.07 ± 0.83^ABa^	14.17 ± 0.55^Cb^
b^*^	T2	14.89 ± 0.01^Cc^	16.34 ± 0.41^BCb^	16.62 ± 0.27^BCb^	18.03 ± 0.01^Aa^
	T3	15.62 ± 0.01^BCb^	16.10 ± 0.55^Cb^	18.03 ± 0.27^Aa^	19.13 ± 0.01^Aa^
	T4	18.28 ± 0.13^Aa^	19.24 ± 0.13^Aa^	19.79 ± 0.13^Aab^	20.58 ± 0.27^Ab^
	T5	15.62 ± 0.27^BCa^	16.34 ± 0.69^BCa^	16.37 ± 0.13^Ca^	16.83 ± 0.41^Ba^

Note

T1: Control, T2: 2% CA, T3: 2% CA +500 ppm AFEOs, T4: 2% CA +1000 ppm AFEOs, and T5: 2% CA +1500 ppm AFEOs. ^a–c^ Different lowercase letters throughout storage indicate significant (*p* < .05) differences ^A–C^ Different capital letters between meat samples indicate significant (*p* < .05) differences.

### 3.6 Microbiological analysis

The results recorded for TVC, coliforms, molds and yeasts count throughout refrigerated period are given in Table 5. At day 1, the TVC for coated meat samples with CA or AFEOs ranged from 5.72 to 5.86 Log CFU/g which had no remarkable differences with control samples (5.88 Log CFU/g). However, at day 12 the TVC of coated samples with CA +AFEOs ranged from 6.89 to 7.61 Log CFU/g which was significantly lower than those of CA‐alone coating (9.69 Log CFU/g) and control (9.80 Log CFU/g). The coated samples with CA +1500 ppm AFEOs displayed acceptable levels of TVC at day 12. According to Chouliara et al. (2008), the acceptable limitation for TVC in fresh poultry meat is 6 Log CFU/g, while control samples exceed this limitation at day 4. The results of TVC are in agreement with those of Jonaidi Jafari et al. (2018), Zhou et al. (2021), and Muhialdin et al. (2020) on chicken fillets, chicken meat, and chicken samples treated with natural coating in combination with plant extracts and EOs.

**TABLE 5:** Evaluation of microbiological count (Log CFU (colony‐forming units)/g) in meat samples treated with calcium‐alginate +AFEOs (*Artemisia fragrance* essential oils) throughout keeping time at 4°C

Microorganisms	Treatments	Storage (day)			
		1	4	8	12
	T1	5.88 ± 0.008^Ab^	6.04 ± 0.01^Ab^	7.95 ± 0.37^Ab^	9.80 ± 0.005^Aa^
TVC	T2	5.86 ± 0.005^Ac^	5.99 ± 0.005^Ac^	7.64 ± 0.003^Ab^	9.69 ± 0.005^Ab^
	T3	5.81 ± 0.011^Ab^	5.94 ± 0.003^Ab^	6.95 ± 0.003^Bab^	7.61 ± 0.01^Ba^
	T4	5.75 ± 0.008^Ab^	5.89 ± 0.003^Ab^	6.63 ± 0.003^Bab^	7.93 ± 0.099^Ba^
	T5	5.72 ± 0.008^Aa^	5.87 ± 0.005^Aa^	6.52 ± 0.014^Ba^	6.89 ± 0.003^Ba^
	T1	2.86 ± 0.008^Ad^	4.99 ± 0.01^Ac^	6.55 ± 0.082^Ab^	9.63 ± 0.017^Aa^
Coliforms	T2	2.83 ± 0.003^ABd^	4.95 ± 0.008^Ac^	6.46 ± 0.003^Bb^	8.97 ± 0.001^Ba^
	T3	2.78 ± 0.005^Bd^	4.87 ± 0.005^Bc^	5.89 ± 0.008^Cb^	7.71 ± 0.014^Ca^
	T4	2.61 ± 0.008^Cd^	4.76 ± 0.003^Cc^	5.54 ± 0.031^Db^	6.70 ± 0.008 Da
	T5	4.64 ± 0.011^Cc^	4.69 ± 0.003^Cc^	5.47 ± 0.020^Db^	6.66 ± 0.001 Da
	T1	1.89 ± 0.003^Ac^	4.21 ± 0.083^Ac^	5.77 ± 0.055^Ab^	8.75 ± 0.014^Aa^
Molds and yeasts	T2	1.91 ± 0.005^Ac^	3.72 ± 0.032^Bc^	5.72 ± 0.011^Ab^	7.91 ± 0.003^Ba^
	T3	1.80 ± 0.008^Ac^	3.90 ± 0.003^ABc^	4.94 ± 0.005^Bb^	6.71 ± 0.003^Ca^
	T4	1.51 ± 0.011^Ac^	3.84 ± 0.005^ABc^	4.72 ± 0.011^Bb^	5.84 ± 0.001 Da
	T5	1.53 ± 0.011^Ac^	3.78 ± 0.003^ABc^	4.69 ± 0.005^Bb^	5.72 ± 0.017 Da

Note

T1: Control, T2: 2% CA, T3: 2% CA +500 ppm AFEOs, T4: 2% CA +1000 ppm AFEOs, and T5: 2% CA +1500 ppm AFEOs. ^a–d^ Different lowercase letters throughout storage indicate significant (*p* < .05) differences ^A–D^ Different capital letters between meat samples indicate significant (*p* < .05) differences.

Coliforms as a hygienic quality indicator (Kunová et al., 2014) increased throughout keeping intervals among all samples, whereas the uncoated samples displayed the highest coliform count. The outcomes of the present study showed the highest antibacterial properties (against TVC, coliforms, and molds and yeasts) for CA +1500 AFEOs coating. Berizi et al. (2018) and Alirezalu et al. (2019) also showed similar results for edible coating in combination with natural plant extracts in rainbow trout and frankfurter‐type sausage, respectively.

Because of the special properties of meat such as optimum pH, adequate supply of nitrogenous substances, and high moisture content, this is known as an ideal medium for the proliferation and growth of fungal species. The chicken meats treated with CA +1000 and 1500 ppm AFEOs showed significantly (*p *< .05) higher inhibitory effects against molds and yeasts throughout storage intervals. The initial count of molds and yeasts ranged from 1.51 to 1.91 Log CFU/g, which increased significantly (*p *< .05) and reached 5.72 and 8.75 Log CFU/g for samples treated with CA +1500 ppm AFEOs and control, respectively, at day 12. Molds and yeasts potentially could grow on surfaces of meats and lead to spoilage and negative effects on organoleptic properties and quality stability. The new coatings acted as a barrier and led to a low oxygen concentration on the surfaces of coated samples. Furthermore, the presence of secondary metabolites in EOs can retard or inhibit the growth of yeasts and molds and bacteria.

### 3.7 Sensory properties

**FIGURE 3:**
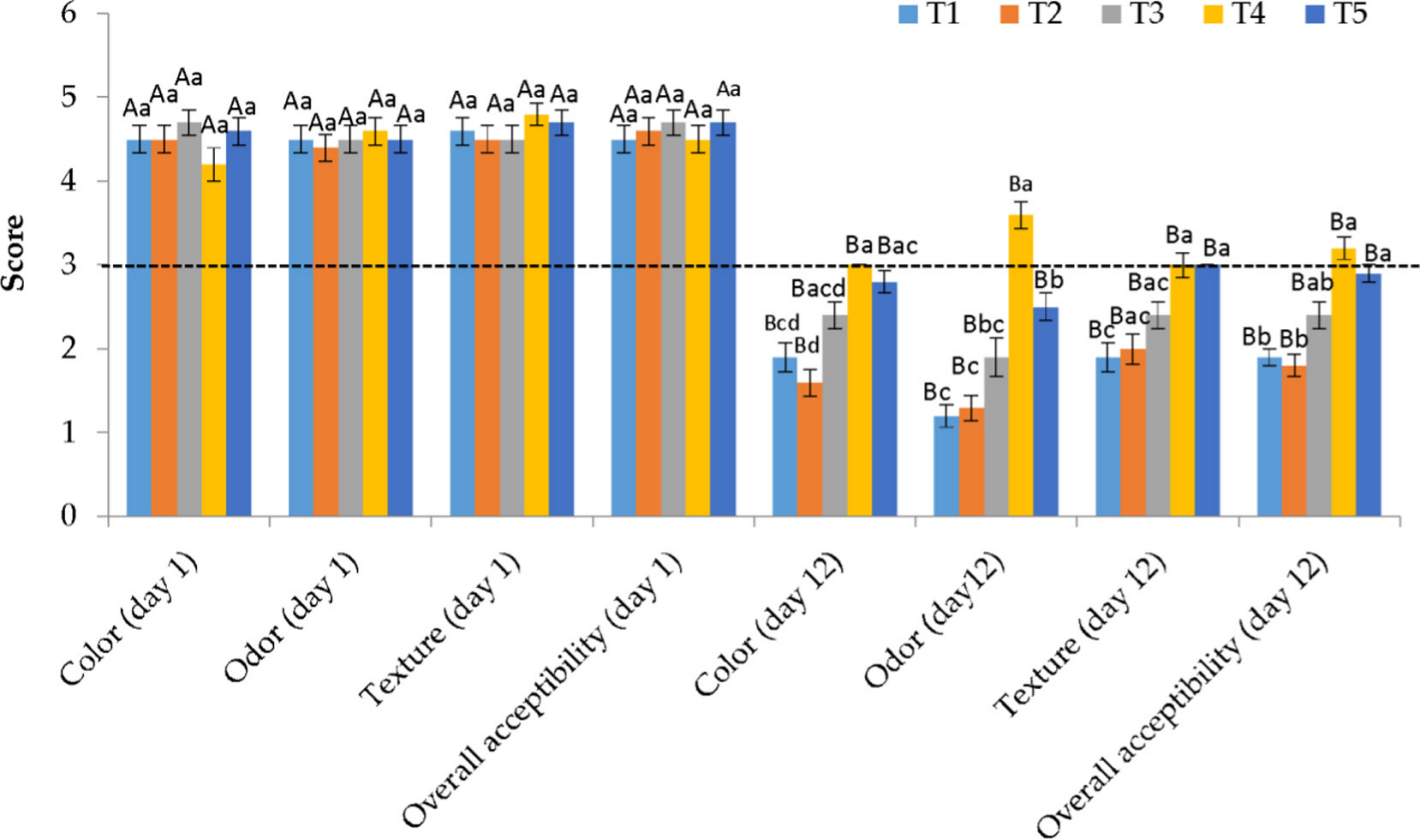
Sensory properties of chicken meat coated with calcium‐alginate +AFEOs (*Artemisia fragrance* essential oils) throughout keeping time at 4°C. T1: Control, T2: 2% CA, T3: 2% CA +500 ppm AFEOs, T4: 2% CA +1000 ppm AFEOs, and T5: 2% CA +1500 ppm AFEOs. ^a–d^ Different lowercase letters throughout storage indicate significant (*p* < .05) differences ^A–B^ Different capital letters between meat samples indicate significant (*p* < .05) differences

Sensory attributes, which highly affected the marketing of products, reflect consumers’ preference and the overall quality of products to a certain extent. With regard to Figure 3, all organoleptic properties including color, odor, texture, and overall acceptability declined considerably throughout the storage period, particularly in uncoated samples. The sensory scores of day 1 indicated that CA +AFEOs had no significant effects on color, odor, texture, and overall acceptability among panelists. The color results revealed that at the end of keeping time, the control samples showed significantly (*p *< .05) lower scores. The texture, odor, and overall acceptability also displayed similar trends. Considerable decreases in the sensory attributes of control samples may be caused by higher oxidation and microbiological growth in comparison with coated samples during the keeping period. The outcomes of the present work indicated that 2% CA +1000 ppm AFEOs could significantly preserve the sensory properties among storage time.

Similar results have been reported in beef fillets coated with Plantago major seed mucilage coating +Anethum *graveolens* essential oils (Alizadeh Behbahani et al., 2017), chitosan coating combined with natural plants’ EOs (Yaghoubi et al., 2021), treated lamb with chitosan coating with *Satureja* plant essential oil (Pabast et al., 2018), and chitosan–gelatin coating +tarragon EOs in pork slices (Zhang et al., 2020).

The outcomes of the present work indicated that the CA coating combined with AFEOs had no adverse effects on the sensory attributes of chicken breast meat. Furthermore, based on the sensory results from microbial growth and chemical reactions, the application of AFEOs in the present work on different aspects of chicken breast meat against microbial spoilage, lipid oxidation, off‐odor, texture, and discoloration throughout the keeping time was acceptable.

## 4 CONCLUSION

The results for chemical composition and microbiological count revealed that CA coating containing AFEOs on chicken breast meat can lead to good quality properties, enhancement of microbiological safety, and improvement of shelf life throughout keeping time. All treatments declined remarkably microbial counts when compared to control. The quality attributes (TBARS, TVB‐N) of treated samples remained within the acceptable range for a longer period. Alginate coating containing 1500 ppm AFEOs had the highest inhibitory effect on lipid and protein oxidations and against microbial growth during keeping time. The outcomes of the present work indicated that the shelf life of chicken breast meat could be remarkably increased by calcium‐alginate coating +1500 ppm AFEOs which can be suggested as potential coating materials. According to results obtained from the commercialization of CA and other coating materials such as chitosan, due to the disproportionate price with the application, these compounds can be used in combination with gelatin protein to coating chicken meat in the meat industry and retail.

## CONFLICT OF INTEREST

The authors reported no potential conflict of interest.

## ETHICAL APPROVAL

This article does not cover any human or animal studies conducted by any of the authors.

## Data Availability

Data are available upon request from the authors.
